# Piperonylic acid alters growth, mineral content accumulation and reactive oxygen species-scavenging capacity in chia seedlings

**DOI:** 10.1093/aobpla/plac025

**Published:** 2022-05-26

**Authors:** Mbukeni Nkomo, Arun Gokul, Roya Ndimba, Mihlali Badiwe, Marshall Keyster, Ashwil Klein

**Affiliations:** Plant Omics Laboratory, Department of Biotechnology, Life Science Building, University of the Western Cape, Robert Sobukwe Road, Bellville 7530, South Africa; Department of Agriculture, University of Zululand, Main Road, KwaDlagezwe 3886, South Africa; Department of Plant Sciences, Qwaqwa Campus, University of the Free State, Phuthadithjaba 9866, South Africa; Radiation Biophysics Division, Ithemba LABS (Laboratory for Accelerator Based Sciences), Nuclear Medicine Department, National Research Foundation, Cape Town 8000, South Africa; Plant Omics Laboratory, Department of Biotechnology, Life Science Building, University of the Western Cape, Robert Sobukwe Road, Bellville 7530, South Africa; Environmental Biotechnology, Department of Biotechnology, Life Science Building, University of the Western Cape, Robert Sobukwe Road, Bellville 7530, South Africa; Centre of Excellence in Food Security, University of the Western Cape, Robert Sobukwe Road, Bellville 7530, South Africa; Plant Omics Laboratory, Department of Biotechnology, Life Science Building, University of the Western Cape, Robert Sobukwe Road, Bellville 7530, South Africa

**Keywords:** Antioxidant enzymes, chia, osmolytes, piperonylic acid, reactive oxygen species

## Abstract

*p*-Coumaric acid synthesis in plants involves the conversion of phenylalanine to *trans*-cinnamic acid via phenylalanine ammonia-lyase (PAL), which is then hydroxylated at the para-position under the action of *trans*-cinnamic acid 4-hydroxylase. Alternatively, some PAL enzymes accept tyrosine as an alternative substrate and convert tyrosine directly to *p-*coumaric acid without the intermediary of *trans*-cinnamic acid. In recent years, the contrasting roles of *p-*coumaric acid in regulating the growth and development of plants have been well-documented. To understand the contribution of *trans*-cinnamic acid 4-hydroxylase activity in *p*-coumaric acid-mediated plant growth, mineral content accumulation and the regulation of reactive oxygen species (ROS), we investigated the effect of piperonylic acid (a *trans*-cinnamic acid 4-hydroxylase inhibitor) on plant growth, essential macroelements, osmolyte content, ROS-induced oxidative damage, antioxidant enzyme activities and phytohormone levels in chia seedlings. Piperonylic acid restricted chia seedling growth by reducing shoot length, fresh weight, leaf area measurements and *p*-coumaric acid content. Apart from sodium, piperonylic acid significantly reduced the accumulation of other essential macroelements (such as K, P, Ca and Mg) relative to the untreated control. Enhanced proline, superoxide, hydrogen peroxide and malondialdehyde contents were observed. The inhibition of *trans*-cinnamic acid 4-hydroxylase activity significantly increased the enzymatic activities of ROS-scavenging enzymes such as superoxide dismutase, ascorbate peroxidase, catalase and guaiacol peroxidase. In addition, piperonylic acid caused a reduction in indole-3-acetic acid and salicylic acid content. In conclusion, the reduction in chia seedling growth in response to piperonylic acid may be attributed to a reduction in *p*-coumaric acid content coupled with elevated ROS-induced oxidative damage, and restricted mineral and phytohormone (indole-3-acetic acid and salicylic) levels.

## Teaser

In this study we investigated the impact of a potent irreversible inhibitor (piperonylic acid) of coumarate-4-hydroxylase, on the physio-biochemical responses of chia seedlings. The results showed that piperonylic acid induced oxidative damage which resulted in restricted plant growth. Piperonylic acid also augmented proline content and activated ROS-scavenging antioxidant enzyme activities which was not sufficient to reduce the ROS-induced damage. In addition, we also observed a reduction in *p*-coumaric acid content, macronutrient accumulation and phytohormone levels which support the hypothesis that *p*-coumaric acid is an important signalling molecule for chia seedling growth.

## Introduction

*p*-Coumaric acid (*p*-CA) is a phenolic compound that is widely distributed in plants and plays a versatile role in modern medicine with antioxidant (Castelluccio *et al.* 1995), cardioprotective (Castelluccio *et al.* 1995), antimicrobial (Cho *et al.* 1998), anti-mutagenic (Ferguson *et al.* 2003), anti-platelet (Luceri *et al.* 2007) and anti-inflammatory (Luceri *et al.* 2004) properties. In addition to the above properties, several other studies support the role of *p*-CA in reducing the rate of seed germination, root length and biomass in different plant species (Patterson 1981; Janovicek *et al.* 1997; Reigosa *et al.* 1999; Baleroni *et al.* 2000; Ng *et al.* 2003; Politycka and Mielcarz 2007). Some studies have suggested that the reduction in plant growth is a coping mechanism of plant defence against phenolic acid-imposed allelochemical stress (Rama Devi and Prasad 1996; Böhm *et al.* 2006; Soares *et al.* 2011). This has led to a general perception that exogenous application of *p-*CA inhibits plant growth and development as observed in leguminous plants (Zanardo *et al.* 2009). However, a recent study by Nkomo *et al.* (2019) showed that exogenous *p-*CA improves chia seedling growth development via the activation of reactive oxygen species (ROS), which modulates antioxidant ROS homeostasis by regulating the levels of compatible osmolytes. In addition, Jones *et al.* (2017) showed that a *p*-CA derivate (caffeic acid) regulated physio-biochemical responses in chia plants via the modulation of antioxidant enzymes, which in turn led to increased chia plant growth. Based on these findings, we hypothesize that different plant species may react differently to exogenous application of phenolic acids (Zanardo *et al.* 2009; Bubna *et al.* 2011; Salvador *et al.* 2013; Nkomo *et al.* 2019).

*p*-Coumaric acid is found in the phenylpropanoid pathway, where it generates an array of secondary metabolites that contribute to all aspects of plant responses towards biotic and abiotic stimuli. The use of inhibitors to inactivate selected steps in the phenylpropanoid pathway may be a useful approach to monitor the physio-biochemical responses in plants and can serve as an alternative method to mutation or transgenic approaches (Schoch *et al.* 2002). Cinnamate 4-hydroxylase (C_4_H) is a member of the structural family of cytochrome P450 heme thiolate proteins, which catalyses the second step of the phenylpropanoid pathway, leading to the synthesis of *p*-CA, lignin, pigments and many defence molecules. The chemical deactivation of C_4_H using a potent inhibitor will block the downstream biosynthesis of *p-*CA. The substrate specificity of several inhibitors of C_4_H has been described (Pierrel *et al.* 1994; Schalk *et al.* 1998; Schoch *et al.* 2002), and the most potent inhibitors reported thus far include 2-hydroxy-1 naphthoate (2HN) and piperonylic acid (PA). Piperonylic acid remains a useful inhibitor as it was demonstrated to inactivate C_4_H with a concentration as low as 10 µM (Schoch *et al.* 2002). As a natural molecule bearing a methylenedioxy function, PA closely mimics the structure of *trans*-cinnamic acid which irreversibly inhibits C_4_H. Most studies focusing on the exogenous application of PA in plants discuss their effects in relation to cell wall lignification and/or salicylic acid (SA) accumulation (Schoch *et al.* 2002; Böhm *et al.* 2006; Ferro *et al.* 2015), whereas the information on plant growth and ROS scavenging remains scant.

A study by Wong *et al.* (2005) investigated the effect of PA on plant growth in response to UV-light exposure and showed that PA restricted *Arabidopsis thaliana* root growth. The reduction in root growth was suggested to be due to *cis*-CA (Åberg 1961; Ugochukwu and Wain 1968) produced when PA blocks the conversion of *trans*-CA to 4-coumaric acid. Another study by Kumar *et al.* (2016) showed that the downregulation of C_4_H enzymatic activity through the RNAi knock-down of AaC_4_H in *Artemisia annua* leads to defects in morphology and anatomy that resulted in stunted growth (Kumar *et al.* 2016). Despite the clear role of ROS molecules as key mediators in controlling plant growth by triggering the activation of many stress-related genes, the reduction of *p*-CA levels on ROS accumulation is unknown. Therefore, the possible effect of PA on ROS homeostasis in plants remains elusive with the only documented case focusing on the growth of keratinocytes (Lee *et al.* 2018). In light of the contradictory role of *p*-CA in plants together with the limited evidence regarding PA-induced restriction in plant growth and ROS-mediated antioxidant changes we investigated the effect of exogenously applied PA on growth, osmolyte content, mineral accumulation, ROS-induced oxidative damage and changes in antioxidant enzyme activities in chia seedlings.

## Materials and Methods

### Plant growth and treatment

Plant growth was investigated in chia seedlings as described by Nkomo *et al.* (2019). Control plants were supplemented with 50 mL of Nitrosol® solution diluted in water (1:300). For treatment with PA, plants were supplemented with Nitrosol® containing 100 µM PA (at 2-day intervals) for a period of 14 days. After 14 days of treatment, chia seedlings were carefully removed from the growth medium to avoid any damage. Subsequently, chia seedling roots were separated from the shoots to prevent erroneous data interpretation caused by possible root damage. Following the method described by Nkomo *et al.* (2019), the shoots from each treatment were scored for length (SL) and fresh weight (FW) and images were captured using a Canon 80D digital camera (lens; Canon EF-S 10–18 mmf/4.5–5.6 IS STM). Leaf area was analysed using ImageJ (https://imagej.nih.gov/ij/docs/pdfs/examples.pdf).

### Measurement of endogenous *p*-CA content

To measure C_4_H enzyme efficiency levels, the levels of endogenous *p*-CA produced by C_4_H enzyme in the shoots of chia seedlings were quantified using reverse-phase high-performance liquid chromatography (RP-HPLC). *p*-Coumaric acid was successfully separated on a Alltima™ C_18_ column (250 mm × 4.6 mm, 5 μm) at 30 °C, using a mixture of acetonitrile (ACN):0.1 % (v/v) acetic acid solution (25:75, v:v) as the mobile phase with detection at 308 nm. The flow rate was 1.0 mL min^−1^, and the injection volume was 20 μL.

### Inductively coupled plasma optical emission spectroscopy analysis

Sample digestion of chia seedling shoots was performed according to Vachirapatama and Jirakiattikul (2008). The concentrations of four essential macroelements (Na, P, K and Mg) was determined using a Varian Vista Pro CCD simultaneous inductively coupled plasma optical emission spectrometer (Varian, Australia) with certified standards (Sigma, St. Louis, MO, USA; TraceCERT®).

### Measurement of superoxide content

Superoxide content (O_2_^−^) in the shoots of chia seedlings was quantified using a method previously described by Gokul *et al.* (2016). Superoxide concentrations were determined by submerging intact seedling shoots in a solution containing 10 mM KCN [to inhibit Cu/Zn superoxide dismutases (SODs)], 10 mM hydrogen peroxide (H_2_O_2_; to inhibit Mn and Cu/Zn SODs), 2 % (m/v) SDS (to inhibit Mn and Fe SODs), 80 μM nitro blue tetrazolium chloride (NBT) (Sigma; powder, for molecular biology) and 50 mM potassium phosphate (pH 7.0). The seedling shoots were incubated for 20 min in the solution after which the seedlings were homogenized (in solution), centrifuged (10 000 × *g* for 5 min) and the supernatant was spectrophotometrically analysed at 600 nm. The superoxide concentration was calculated using the NBT extinction coefficient of 12.8 mM cm^−1^.

### Protein extraction for biochemical analysis

Chia shoots from all treatments were harvested and ground into a fine powder using liquid nitrogen. Shoots (0.1 g) were homogenized in 1 mL of 6 % (w/v) trichloroacetic acid (TCA) for analysis of H_2_O_2_ content and lipid peroxidation or in 1 mL of homogenizing PVP buffer (40 mM K_2_HPO_4_ at pH 7.4; 1 mM EDTA; 5 % PVP MW = 40 000; 5 % glycerol in distilled H_2_O) for the measurement and detection of total SOD, ascorbate peroxidase (APX), catalase (CAT) and guaiacol peroxidase (POD) enzymatic activity. The protein concentrations were determined using the RC DC Protein Assay Kit 11 (Bio-Rad Laboratories).

### Measurement of H_2_O_2_ content

Hydrogen peroxide content was determined based on a method previously described by Velikova *et al.* (2000). The reaction mixture contained 75 μL of the TCA extract, 5 mM K_2_HPO_4_, pH 5.0 and 0.5 M KI. Samples were incubated at 25 °C for 20 min and absorbance readings of the samples were recorded at 390 nm. Hydrogen peroxide content was calculated using a standard curve based on the absorbance (A390 nm) of H_2_O_2_ standards.

### Determination of malondialdehyde content

The extent of lipid peroxidation (malondialdehyde; MDA) in the shoots of chia seedlings was quantified as described by Egbichi *et al.* (2013). Chia shoots (100 mg) were ground into a fine powder in liquid nitrogen. The tissue was homogenized in 400 μL of cold 5 % (w/v) TCA. The homogenate was centrifuged at 12 000 × *g* for 30 min at 4 °C. Aliquots (100 μL) of the supernatant were mixed with 400 μL of 0.5 % TBA (prepared in 20 % TCA). The mixture was incubated at 95 °C for 30 min and the reaction was stopped by placing the mixture on ice for 5 min. The mixture was further centrifuged at 12 000 × *g* for 5 min at 4 °C. The absorbance of the supernatant was measured at 532 and 600 nm. After subtracting the non-specific absorbance (A600 nm), the MDA concentration was determined by its extinction coefficient of 155 mM^−1^ cm^−1^ and expressed as nmol g^−1^ of fresh weight.

### Measurement and detection of SOD activity

The spectrophotometric SOD assay was determined from a modified method by Beauchamp and Fridovich (1971). For this spectrophotometric method, 190 μL of the assay buffer (50 mM K_2_HPO_4_, pH 7.8, 0.1 mM EDTA, 10 mM methionine, 5 μM riboflavin, 0.1 mM NBT) and 10 μL of shoot extracts were mixed. The mixture was incubated at room temperature for 20 min on a fluorescent light box and absorbance readings at 560 nm were recorded. Superoxide dismutase activity was calculated based on the amount of enzyme that was required to cause a 50 % decrease in the reduction of NBT to blue formazan.

### Measurement of APX activity

Ascorbate peroxidase activity in the shoots of chia seedlings was measured using a modified method previously described by Asada (1992). Each reaction contained 10 μL PVP protein extract and 180 μL of APX assay buffer (50 mM K_2_HPO_4_ at pH 7.0; 0.2 mM EDTA; buffer 0.25 mM ascorbic acid in distilled H_2_O) in a final volume of 190 μL. The reaction was initiated with the addition of 10 μL H_2_O_2_ (90 μM), and the absorbance measured at 290 nm. Ascorbate peroxidase activity was calculated using the extinction coefficient of 2.8 mM^−1^ cm^−1^.

### Measurement of CAT activity

Catalase activity was determined by measuring the H_2_O_2_ consumption at 240 nm according to a modification of the method of Aebi (1984). A reaction mixture was prepared containing 50 mM K_2_HPO_4_ (pH 7.0), 0.5 mM EDTA and 20 μg protein extract. The CAT reaction was initiated by addition of 10 mM H_2_O_2_ and absorbance was measured immediately. The extinction coefficient of H_2_O_2_ (43.6 M^−1^ cm^−1^) was used to calculate the enzyme activity and expressed as µmol·min^−1^·mg^−1^ of protein.

### Measurement of total POD activity

Guaiacol peroxidase activity in the shoots of chia plants was estimated using a modified method previously described by Pitel and Cheliak (1986). The reaction mixture consisted of 100 mM Na-acetate (pH 5.3), 37 mM guaiacol, 10.3 mM H_2_O_2_ and 100 μL of PVP protein extract in a final volume of 3 mL. The reaction mixture was incubated at 30 °C for 15 min and absorbance was recorded at 436 nm. Guaiacol peroxidase activity was calculated using the extinction coefficient of 26.6 mM^−1^ cm^−1^.

### Determination of proline content

Total free proline content in the shoots of chia plants was estimated using a modified method described by Nxele *et al.* (2017). Fresh shoot material from each treatment (0.1 g) was homogenized in 500 μL of 3 % (w/v) sulphosalicylic acid using a mortar and pestle. About 200 μL of each homogenate was mixed with 200 μL of glacial acetic acid to which 200 μL of ninhydrin was added. The reaction mixture was boiled in a water bath at 100 °C for 30 min and immediately cooled in an ice bath. After cooling, 400 μL of toluene was added to the reaction mixture. After thorough mixing, the chromophore containing toluene was separated and absorbance of the red colour that developed was read at 520 nm against a toluene blank using a FLUOstar Omega UV-visible spectrophotometer (BMG LabTech GmbH, Ortenberg, Germany).

### Quantification of phytohormone content

Quantification of plant hormones [indole-3-acetic acid (IAA), salycilic acid (SA) and jasmonic acid (JA)] was conducted using the method of Šimura *et al.* (2018) with slight modifications. Briefly, chia seedling shoots were flash-frozen in liquid nitrogen and ground to a fine powder. Samples (200 mg) were extracted with 1.6 mL ice-cold 50 % (v/v) ACN with vibration milling and sonication for 10 min. The samples were extracted with a benchtop mixer for 30 min at 4 °C following by centrifugation for 10 min (35 000 × *g* at 4 °C). The supernatants were purified using Oasis HLB RP columns (Waters) washed with 1 mL of 100 % MeOH and 1 mL of deionized water, then equilibrated with 50 % aqueous (v/v) ACN. The flow-through fraction was collected by elution with 1 mL of 30 % (v/v) ACN and the samples were dried under a stream of nitrogen. The dried samples were dissolved in 40 µL of 30 % (v/v) ACN and 10 µL of each sample was injected into the UHPLC-ESI-MS/MS system and the different hormones were quantified using internal standards for IAA, SA and JA (Šimura *et al.* 2018).

### Statistical analysis

All experiments described were performed six times independently. For measurement of plant growth parameters (shoot height and shoot fresh weight) and superoxide content, 30 individual chia seedlings per treatment were analysed. For all other experiments, 50 chia seedling shoots were homogenized per treatment. For statistical analysis, the one-way analysis of variance test was used for all data, and the means (for six independent experiments) were compared according to the Tukey–Kramer test at 5 % level of significance, using GraphPad Prism 5.03 software.

## Results

### Inhibition of *p*-CA restricts chia plant growth and *p*-CA content

**Figure 1.**
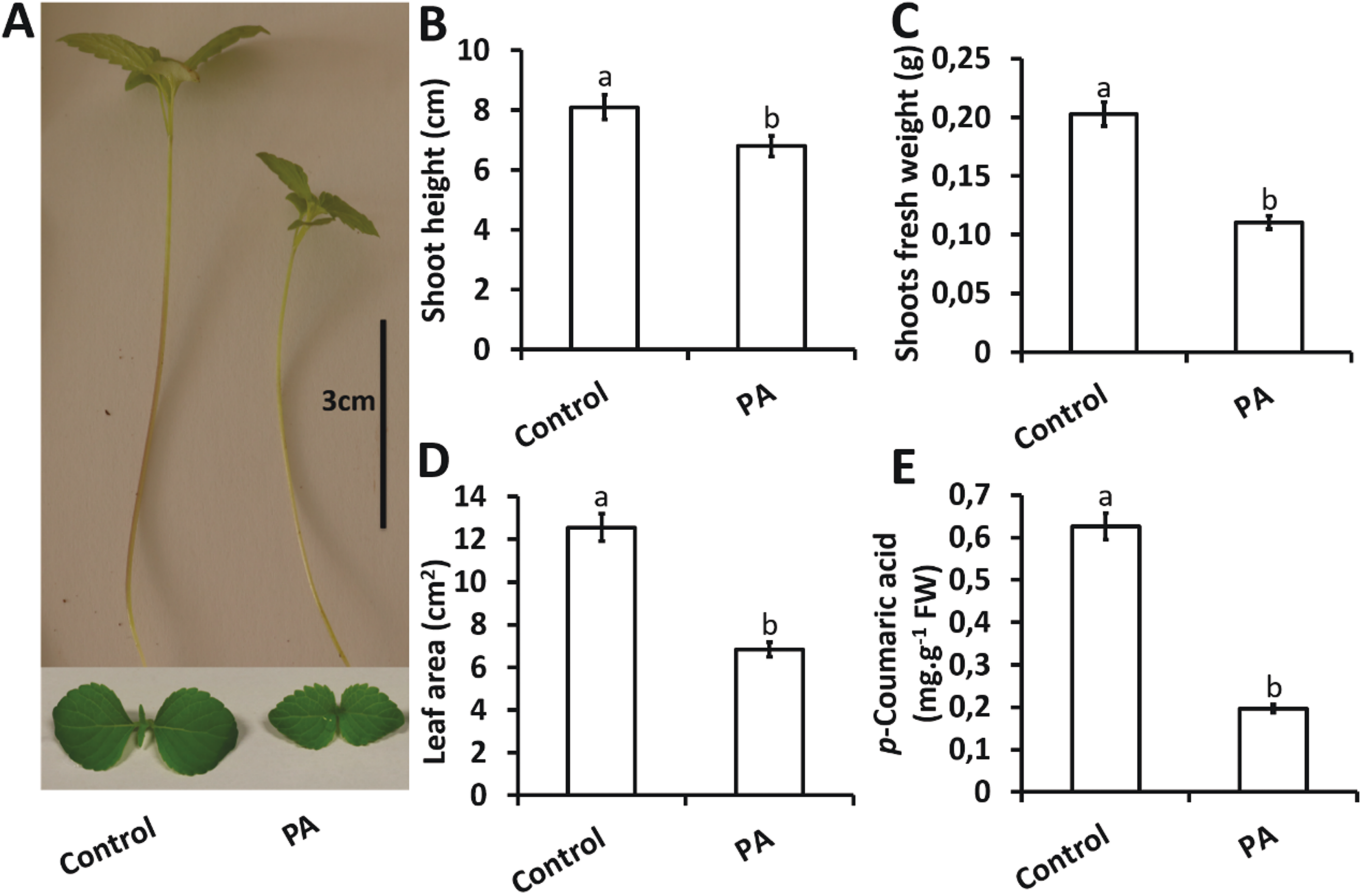
Exogenous PA restricts chia seedling growth and *p*-CA content. Representative chia seedling shoots under control and PA treatments (A), shoot height (B), fresh weight (C), leaf area (D) and measurement of *p*-CA (E). Data represent the mean (±SE) from six independent experiments. Different letters above the error bars indicate means that are significantly different at the 5 % level of significance.

Plants treated with PA exhibited a loss in shoot height (16 %) and fresh weight (46 %) when related to control plants (Fig. 1A–C). A similar trend was observed for the leaf area measurement in PA treatment compared to the control (Fig. 1D). The leaf area of chia seedlings treated with PA was reduced by 46 % relative to the control (Fig. 1D). A direct relationship exists between C_4_H activity and *p*-CA production; therefore, quantifying the level of *p*-CA in the shoots of chia seedlings serves as a good indicator of C_4_H activity. The results showed that exogenous PA reduced C_4_H activity (as seen for *p*-CA content levels) in the shoots of chia seedlings by 68 %, relative to the control (Fig. 1E).

### A survey of essential macroelements in response to exogenous PA

Changes in mineral content were monitored in the shoots of chia seedlings after treatment with PA. The mineral contents of five essential macroelements measured in chia seedlings are shown in Table 1, and the values are expressed as mg g^−1^ fresh weight of plant material. The macroelements analysed included sodium (Na), magnesium (Mg), phosphorus (P), potassium (K) and calcium (Ca). For the mineral content analysis, we expressed our results relative to the controls and used a red arrow (indicates a decrease) and blue arrow (indicates an increase) to express the abundance of essential macroelements. The macroelement Na was increased by 19 % in response to PA when compared to the control (Table 1). Interestingly, the rest of the essential macroelements showed a significant reduction in content in response to PA when compared to the control. Potassium (K) content was reduced by 64 % in the PA treatment relative to the control. Both phosphorus (P) and magnesium (Mg) contents were reduced by 56 % when compared to their respective control. Finally, calcium (Ca) content was decreased by 53 %, when compared to the control.

**Table 1.** Content of essential macroelements in the shoots of chia seedlings. Macroelements data expressed in mg·g^−1^ FW, presented by the means ± SE (*n* = 3). The blue arrow represents an increase in macroelements, while the red arrow represents a decrease when comparing the PA treatment to the control.

Minerals	Mineral relative content (mg·g^−1^ FW)		Class
	Control	PA	
Na	0.057 ± 0.004	0.068 ± 0.005	Essential macroelements
K	4.513 ± 0.0068	1.609 ± 0.048	
P	0.329 ± 0.002	0.146 ± 0.005	
Mg	0.387 ± 0.006	0.169 ± 0.006	
Ca	0.497 ± 0.002	0.231 ± 0.006	

### PA increases ROS accumulation and the extent of lipid peroxidation

**Figure 2.**
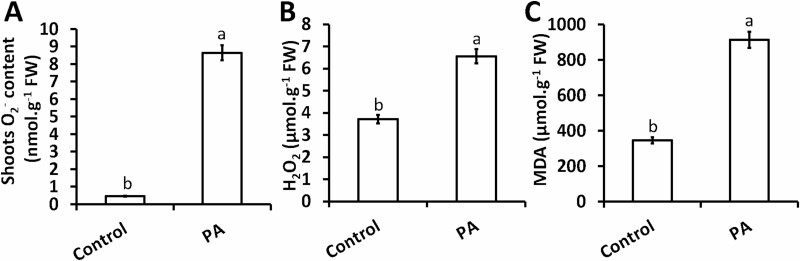
The influence of PA on superoxide content (A), H_2_O_2_ content (B) and MDA content (C). The O_2_^−^ content was measured using freshly harvested shoot material from chia seedlings. Data represent the mean (± SE) of six independent experiments. Means with different letters are significantly different from each other (*P* < 0.05).

The impact of the inhibition of *p*-CA production (as a consequence of PA treatment) on ROS marker accumulation and ROS-induced oxidative damage was measured. Exogenous application of PA significantly increased superoxide content by 1755 % in the shoots of chia seedlings when related to the control seedlings (Fig. 2A). A similar trend was observed for H_2_O_2_ content although not to the same extent as was observed for superoxide content. Chia seedlings treated with PA increased H_2_O_2_ content in the shoots by 77 % when related to the control (Fig. 2B). The increase in ROS biomarkers resulted in a significant increase in oxidative damage manifested as enhanced levels of MDA. The MDA content in PA-treated plants was increased by 164 % relative to that of the control plants (Fig. 2C).

### Exogenous PA augments proline and total SOD in the shoots of chia seedlings

**Figure 3.**
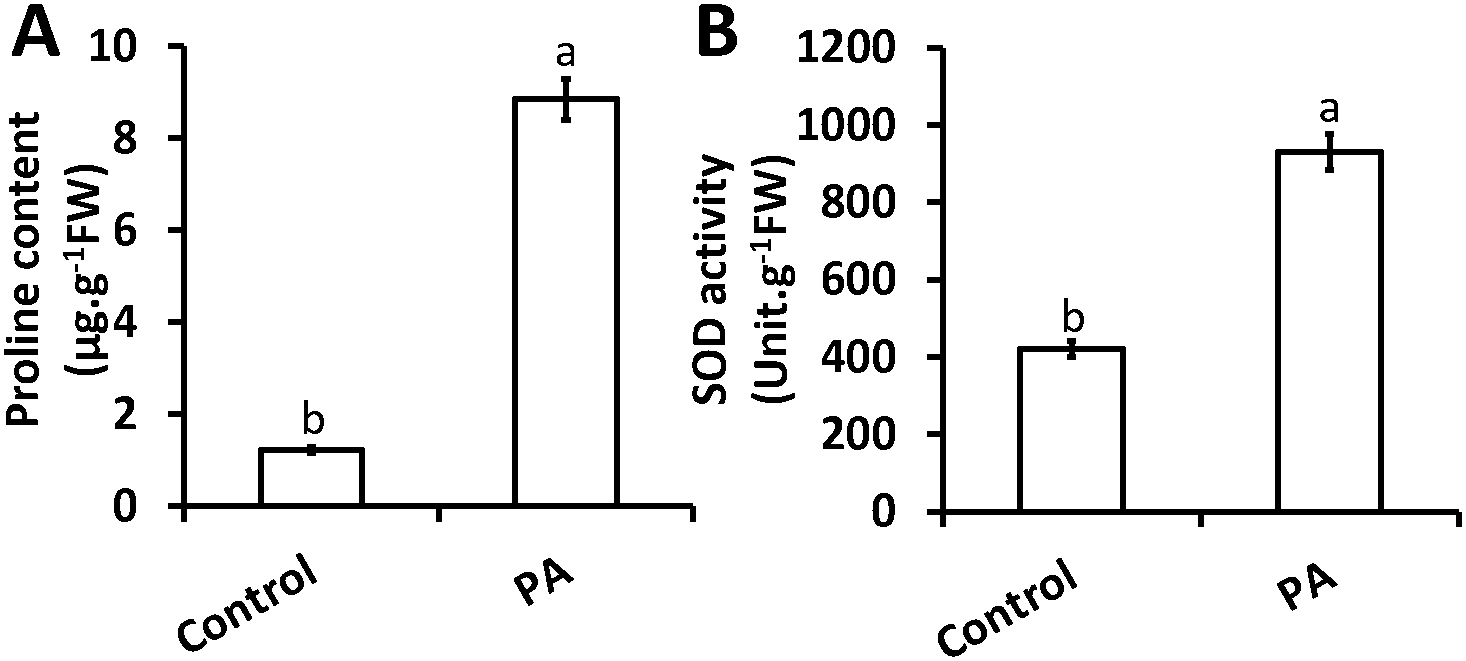
Piperonylic acid increases proline content (A) and total SOD activity (B) in chia seedling shoots. Data represent the mean (±SE) of six independent experiments. Different letters represent statistical significance at *P* < 0.05 according to the Tukey–Kramer test.

It is well known that the accumulation of ROS molecules triggers a cascade of events that ultimately leads to the degradation of lipid membranes (known as lipid peroxidation). Osmolytes such as proline play a highly beneficial role in plants exposed to various stress conditions. Besides acting as an excellent osmolyte, proline helps plants minimize ROS-induced oxidative damage by means of direct ROS scavenging. Here, we illustrate a direct relationship between ROS accumulation (Fig. 2A and B) and increased proline content in chia seedlings treated with PA (Fig. 3A). A significant increase in proline content (622 %) was observed in the shoots of chia seedlings in response to exogenous PA relative to the control plants (Fig. 3A). In light of the augmented levels of superoxide observed in chia shoots treated with PA (Fig. 2A), changes in total SOD activity (superoxide scavenging antioxidant enzyme) in the same tissue were measured. The results showed that exogenous PA also increased total SOD activity (121 %) to levels that were significantly higher than those in control plants (Fig. 3B).

### Changes in H_2_O_2_-scavenging antioxidant enzyme activities in response to PA

**Figure 4.**
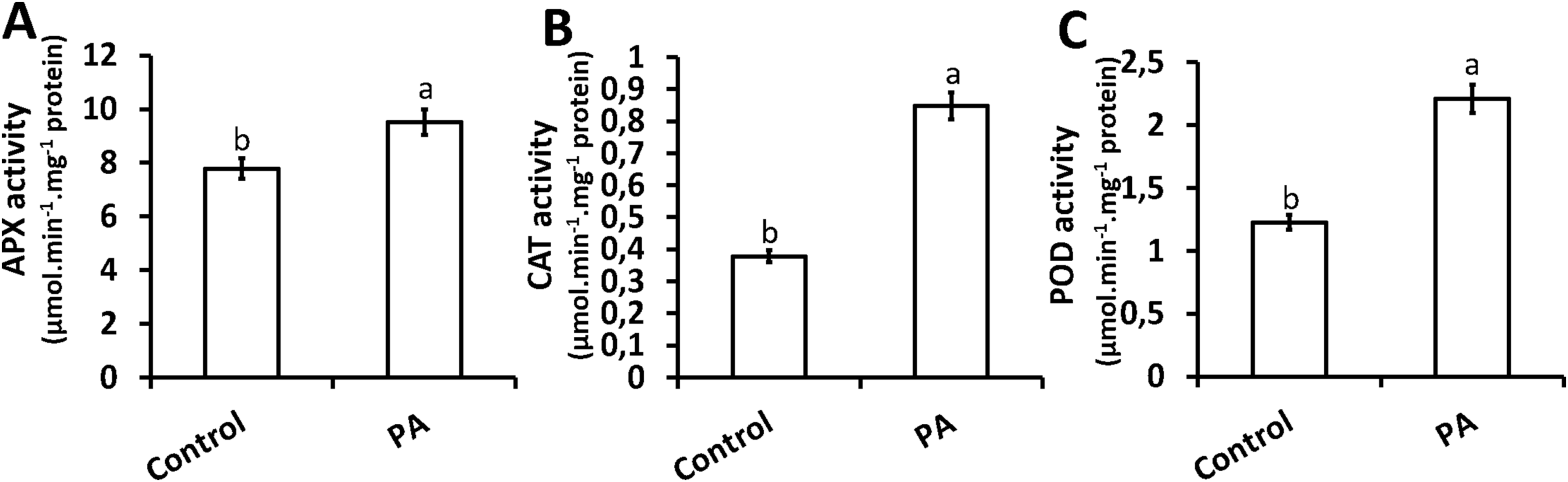
Changes in shoot APX (A), CAT (B) and POD (C) activities in response to exogenous PA. Data represent the mean (±SE) of six independent experiments. Different letters represent statistical significance at *P* < 0.05 according to the Tukey–Kramer test.

Alteration in the activity of antioxidant enzymes in response to ROS-induced oxidative damage is well-documented in different plant species, and there is also evidence (albeit minimal) that such responses are modulated by phenolic acids, including *p-*CA. However, information on the modulation of these antioxidant responses when *p-*CA is inhibited remains elusive. In light of the increase in H_2_O_2_ content (Fig. 4B) we investigated the effect of PA (*p-*CA inhibitor) on the enzymatic activities of some H_2_O_2_-scavenging enzymes including peroxidases such as APX, POD and CAT. The results showed that exogenous PA increased the total enzymatic activities of all three antioxidants relative to their respective controls (Fig. 4).

Ascorbate peroxidase activity in the shoots showed an increase of 22 % in response to treatment with PA when compared to the control (Fig. 4A). Similar trends were observed for enzymatic activities of CAT and POD in response to PA although their increase in activity was significantly higher than was observed for APX when compared to their respective controls. The enzymatic activity of CAT in the shoots of chia seedlings was increased by 124 % in response to PA when compared to the untreated controls (Fig. 4B). Guaiacol peroxidase activity in the shoots of chia seedlings was augmented by 80 % in response to treatment with PA relative to the control (Fig. 4C).

### Exogenous PA alters phytohormone contents in chia seedling shoots

The essential role of plant hormones in developmental processes is well-documented. Here we measured the impact of PA on the levels of IAA, SA and JA. Exogenous PA caused a reduction in IAA and SA, whereas an increase was observed for JA when compared to the control (Table 2). Indole-3-acetic acid content in the shoots of chia seedlings was reduced by 34 %, whereas a 37 % decrease in SA content was observed in the PA treatment when compared to the control. In contrast, exogenous PA increased JA content by 59 % when compared to the control.

**Table 2.** Changes in phytohormone contents in the shoots of chia seedlings in response to exogenous PA. Phytohormone data expressed in µg·mg^−1^ FW, presented as the means ± SE (*n* = 4).

Phytohormones	Phytohormones content (µg·mg^−1^ FW)	
	Control	PA
IAA	56.69 ± 7.87	37.18 ± 4.46
SA	4720.78 ± 458.78	2977.40 ± 406.71

## Discussion

*p*-Coumaric acid is one of the key compounds in the phenylpropanoid metabolism pathway, which is mostly involved in promoting plant growth and survival. The present study showed that inhibition of endogenous *p*-CA (using PA) significantly affected chia seedling growth. Furthermore, this reduction in plant growth was accompanied by biochemical alterations of phytohormones, essential macroelements and ROS-signalling homeostasis. Our current study was based on the findings of Nkomo *et al.* (2019), who showed that exogenous application of *p-*CA enhanced chia seedling growth. To our knowledge, this was the first study to show a positive effect of exogenous *p-*CA on plant growth. Hence, we attempted to establish a link or prove the role of *p-*CA as a plant growth-signalling molecule by inhibiting the enzymatic activity of the enzyme (C_4_H) responsible for its biosynthesis. This was achieved through the use of a selective inhibitor, PA, which inhibits the production of endogenous *p-*CA by blocking *trans*-C_4_H activity (Soares *et al.* 2011).

The underlying idea behind this study was to demonstrate that if *p-*CA improved plant growth, then inhibition of endogenous *p-*CA could perturb the growth and development of chia seedlings. We analysed the efficiency of the C_4_H enzyme in producing endogenous *p-*CA, and noted that exogenous PA significantly reduced *p-*CA content in the shoots of chia seedlings (leading to a 69 % irreversible reduction) relative to the untreated control (Fig. 1E). This was in line with the findings of Schalk *et al.* (1998), who showed PA to be an efficient inhibitor (leading to a 58 % irreversible reduction) of C_4_H compared to other tested inhibitors. In addition to the reduction in *p*-CA content we observed a significant reduction in plant growth as shown by reduced shoot length, fresh weight and leaf area measurement in response to exogenous PA relative to the control (Fig. 1). The decrease in *p*-CA accumulation together with a reduction in growth and biomass suggests a direct link between *p*-CA biosynthesis and plant growth. Several papers have described how various phenylpropanoid pathway mutants cause seedling growth arrest (Hoffmann *et al.* 2004; Schilmiller *et al.* 2009; Bonawitz and Chapple 2013; De Meester *et al.* 2018). However, only a few studies using PA as a specific elicitor and describing its impact on plant root growth and phytohormone (auxin) levels have been documented (Wong *et al.* 2005; El Houari *et al.* 2021a, b). In addition to root suppression, El Houari and colleagues detected a considerable alteration in plant growth and leaf development, which is consistent with the results observed in our study (Fig. 1).

Phytohormones such as IAA and SA are plant growth regulators that control a number of agriculturally important processes, including plant development and survival. As a result, it is crucial to determine whether phytohormones are linked to the growth-inhibition effect in response to exogenous PA reported in this study (Fig. 1). According to the findings recorded in Table 2, exogenous PA differentially altered three growth regulators, with a significant reduction observed for IAA and SA coupled with an increase for JA (Table 2).

Indole-3-acetic acid is a derivative of indole that is involved in promoting cell division and/or cell expansion required for optimal growth. Taiz and Zeiger (1998) showed that an increase in IAA resulted in an increase in the growth of sunflower plants. In our study we showed that IAA levels were reduced in response to exogenous PA, which correlated with a reduction in plant growth (Fig. 1). This observation was supported by a similar study of El Houari *et al.* (2021a) who showed that exogenous PA (at a final concentration of 50 µM) reduced IAA levels, which resulted in decreased root growth of *A. thaliana*. The decrease in IAA was most likely due to PA interfering with auxin conjugation by competing for the same enzyme, causing cellular auxin levels to fluctuate (El Houari *et al.* 2021a). It is therefore evident that the reduction in plant growth (Fig. 1) may be linked to a reduction in IAA (Table 2), which is supported by a few previous studies (Tian *et al.* 2008; Wang *et al.* 2016).

Salicylic acid is an endogenous plant growth regulator that acts as a non-enzymatic antioxidant and has an impact on a number of physiological and biochemical processes (Grown 2012). It has been proposed that some plant species can synthesize SA from cinnamate by an unknown mechanism (Schoch *et al.* 2002), which suggests a link between the phenylpropanoid pathway and SA biosynthesis. Schoch *et al.* (2002) did not observe any changes in SA accumulation in tobacco cells. However, we observed a reduction in SA levels in response to PA treatment. It is worth noting that Schoch *et al.* (2002) used a concentration of PA that was 10-fold less compared to the 100 µM used in our study. This could explain why they did not observe a change in SA levels as opposed to the reduction we observed in our study (Table 2).

Inhibition of plant growth is caused by a plethora of biological processes, with ROS-induced damage and changes in phytohormone levels being the most dominant in literature. While evidence about the modulation of ROS-induced oxidative stress using phenolic acids is slowly emerging (Klein *et al.* 2015; Jones *et al.* 2017) little information is known about the mechanisms by which phenolic acids regulate these ROS molecules. Here, we analysed the effect of PA on ROS accumulation and the extent of lipid peroxidation (measured as MDA) in the shoots of chia seedlings. The role of PA in ROS accumulation in plants remains unclear and the only report found in the literature suggests that treatment with 100 µM PA does not increase ROS accumulation (Lee *et al.* 2018). This seems to contradict the observation made in this study, where we showed that PA at a final concentration of 100 μM increased ROS accumulation (as seen for O_2_^−^ and H_2_O_2_ levels) in the shoots of chia seedlings (Fig. 2A and B). However, our results are consistent with those obtained by Desmedt and colleagues, who showed that PA-treated plants experience a transient accumulation of ROS that begins within 1 h of treatment (Desmedt *et al.* 2021). The observed increase in ROS molecules (Fig. 2A and B), and consequent increase in MDA content (Fig. 2C), seems to highlight a direct correlation between ROS accumulation and damage to membrane lipids (Keyster *et al.* 2012, 2013; Nxele *et al.* 2017). In order to counter the adverse effects of ROS-induced oxidative stress, plants exhibit a variety of adaptive strategies including the accumulation of compatible solutes, such as proline, and the activation of ROS-scavenging antioxidant enzymes.

Proline plays an essential role in osmotic adjustment and stabilization of enzymes involved in ROS scavenging (Mittler 2002; Yancey 2005; Slama *et al.* 2015). This in turn helps re-establish a cellular redox balance through suppression of ROS production. Although an existing body of knowledge is available on the effects of phenolic compounds on proline accumulation (Silva *et al.* 2018; Linić *et al.* 2019; Wang *et al.* 2019), there seems to be lack of data or literature on the effect of PA on proline content/accumulation. The role of proline in scavenging ROS molecules in plants has been well-documented (Mittler 2002; Slama *et al.* 2015; Linić *et al.* 2019). Nkomo *et al.* (2019) showed that increased proline accumulation in *p*-CA treatment was essential for scavenging O_2_^−^ radicals. On the contrary, the increased levels of proline (Fig. 3A) in response to PA observed in this study did not restrict superoxide accumulation (Fig. 2A). We suggest that increased proline in response to PA treatment did not scavenge ROS molecules but rather acted as an indicator of stress. The phenomenon observed here is supported by the dual role of proline in plants that is well-documented (Mittler 2002; Linić *et al.* 2019).

The present study showed that PA inhibits the C_4_H enzyme, which triggered some of the key enzymes of the antioxidant defence system (SOD, APX, POD and CAT) as a result of an imbalance in ROS homeostasis in chia seedlings. Superoxide dismutase is considered as a first-line defence system against ROS as it plays an essential role in O_2_^−^ detoxification, producing H_2_O_2_ as a by-product (Foyer and Noctor 2005). In this study, we observed the enhanced conversion of O_2_^−^ to H_2_O_2_ due to the increase in SOD enzymatic activity in response to inhibition of endogenous *p*-CA by PA treatment (Fig. 3B). There is also an increasing body of evidence suggesting that higher levels of H_2_O_2_ lead to oxidative damage in plants (Keyster *et al.* 2012, 2013). To resist oxidative damage, antioxidant enzymes including APX, CAT and POD have been associated with H_2_O_2_ scavenging (Keyster *et al.* 2012; Klein *et al.* 2015; Jones *et al.* 2017). According to Pandey *et al.* (2015), APX is a key enzyme within the ascorbate–glutathione (Halliwell–Asada) pathway that converts H_2_O_2_ to H_2_O and O_2_^−^, using ascorbate (AsA) as an electron donor. In this study, we showed that APX activity increased when endogenous *p-*CA was inhibited using PA (Fig. 4A). This similar trend was also observed for another peroxidase (POD) that utilizes guaiacol instead of ascorbate (Fig. 4C). In the current study a higher level of CAT enzymatic activity was also observed; CAT is known to dismutase H_2_O_2_ molecules without the use of any substrate. It is noteworthy that the effect of PA on antioxidant enzyme activities remains unclear and to our knowledge this study is the first of its kind to show the influence of exogenous PA on enzymatic activities (SOD, APX, CAT and POD) in plant species. We therefore can only speculate that the increase in H_2_O_2_ scavenging enzymes was not high enough to cause an appreciable decrease in H_2_O_2_, which led to an increase in lipid peroxidation.

Plant growth and development are altered by a multitude of biochemical mechanisms, including regulation of ROS homeostasis (Keyster *et al.* 2013; Jones *et al.* 2017; Nxele *et al.* 2017; Nkomo *et al.* 2019) and alteration in essential mineral contents (Munns and Termaat 1986; Cakmak and Kirkby 2008; Kaya 2016). To date, there is no direct link between the effect of PA on essential macroelements (Na, K, P, Mg and Ca) in plants. Our results showed that exogenous PA increased Na accumulation, which was accompanied by a decrease in the other essential macroelements (K, P, Mg and Ca) (Table 1). According to Nazar *et al.* (2015) an increase in endogenous SA concentration led to a decrease in Na content, whereas Latef *et al.* (2021) observed that an increase in IAA concentration did not alter Na content. Therefore, we suggest that the increase in Na content was a result of the decrease in SA levels caused by the exogenous application of PA on chia seedlings. Furthermore, we observed a reduction in K content in response to PA application. According to Marschner (1995), an increase in Na content led to a decrease in K content. Therefore, we suggest that PA application increased the Na content which led to a decrease in K content in chia seedlings. Calcium (Ca) is an essential macroelement that has been reported to not only play a role in plant development (Hasan 2002), but also in controlling K selective channels (Song and Fujiyama 1996). Parallel to the reduction in K we also observed a reduction in Ca. These observations are in line with those of Raven *et al.* (1992), who showed that low levels of Ca promote selectivity for Na over K due to the alteration in the Na/K channels. However, given the lack of a comprehensive genomic and/or transcriptomic data set for chia, investigating the expression levels of genes coding for Na/K transporters remains a challenge. Nonetheless, the selectivity of Na over K caused by Ca could be another reason why we observed an increase in Na parallel to a reduction in Ca and K under PA treatment. We also observed that Na accumulation was accompanied by Mg reduction, which is consistent with an observation made by Loupassaki *et al.* (2002). In addition, we observed a decrease in P content in response to PA treatment, which could be the result of Na accumulation (Su *et al.* 2022). In addition to mineral antagonism and synergism caused by PA in our study, we hypothesize that SA and IAA could also regulate the macroelements. Belkhadi *et al.* (2010) observed an increase in K, Mg and Ca contents and Abbasi *et al.* (2020) observed an increase in P content when plants were treated with SA, respectively. When plants were treated with IAA, Latef *et al.* (2021) observed an increase in K, Mg and Ca contents and Abd El-Samad (2013) observed an increase in P content, respectively. Therefore, the decrease in K, Mg, Ca and P in our study could also be a result of the decrease in SA and IAA levels.

In conclusion, we have demonstrated that exogenous application of PA (100 µM) reduced *p*-CA levels in chia seedlings. As a consequence, all downstream physiological and biochemical processes in chia seedlings evaluated in this study were altered by the exogenous application of PA. Here we have shown that PA application reduced plant growth and the levels of essential macroelements (K, Mg, Ca and P) and phytohormones (SA and IAA). Chia seedlings treated with PA also displayed an increase in Na content, ROS levels (O_2_^−^, H_2_O_2_), MDA content, proline and ROS-scavenging enzyme (SOD, APX, CAT, POD) activities.

The observed effects of PA application could be the result of *p*-CA reduction, which negatively affected plant growth and essential biochemical processes. In a previous study by Nkomo *et al.* (2019) it was shown that exogenous *p*-CA improved plant growth, and therefore reducing *p*-CA levels (as shown in this study) supports the notion that *p*-CA is an important molecule for chia seedling growth. The lack of a comprehensive chia genome curtails opportunities to investigate the expression levels of genes (phytohormones, transporters, osmolytes and antioxidants) affected by PA. Therefore, future work should focus on the use of omics tools (transcriptomics and proteomics) to identify crucial molecular targets of PA to improve our understanding of *p*-CA signalling in chia plants.

## Data Availability

The authors confirm that the data that support the findings of this study are available within the article.

## Notes

**Phenome, Genome & Environment. Chief Editor**: Colleen Doherty
